# Population-based prevalence of congenital defects in a routine sentinel-site based surveillance system in the Western Cape, South Africa

**DOI:** 10.1002/bdr2.2388

**Published:** 2024-08

**Authors:** Emma Kalk, Alexa Heekes, Diane Lavies, Lizel Jacobs, Careni Spencer, Alison Boutall, Ayesha Osman, Chantal Stewart, Mary-Ann Davies, Anika van Niekerk, Karen Fieggen, Andrew Boulle, Ushma Mehta

**Affiliations:** Centre for Infectious Disease Epidemiology & Research, School of Public Health, University of Cape Town; Sub-Saharan African Congenital Anomalies Network; Centre for Infectious Disease Epidemiology & Research, School of Public Health, University of Cape Town; Health Intelligence Directorate, Western Cape Government Health; Centre for Infectious Disease Epidemiology & Research, School of Public Health, University of Cape Town; Sub-Saharan African Congenital Anomalies Network; Centre for Infectious Disease Epidemiology & Research, School of Public Health, University of Cape Town; Sub-Saharan African Congenital Anomalies Network; Division of Human Genetics, Department of Medicine, University of Cape Town & Groote Schuur Hospital; Department of Obstetrics & Gynaecology, University of Cape Town & Groote Schuur Hospital; Department of Obstetrics & Gynaecology, University of Cape Town & Groote Schuur Hospital; Department of Obstetrics & Gynaecology, University of Cape Town & Mowbray Maternity Hospital; Centre for Infectious Disease Epidemiology & Research, School of Public Health, University of Cape Town; Health Intelligence Directorate, Western Cape Government Health; Department of Paediatrics & Child Health, University of Cape Town & Mowbray Maternity Hospital; Division of Human Genetics, Department of Medicine, University of Cape Town & Groote Schuur Hospital; Sub-Saharan African Congenital Anomalies Network; Centre for Infectious Disease Epidemiology & Research, School of Public Health, University of Cape Town; Health Intelligence Directorate, Western Cape Government Health; Centre for Infectious Disease Epidemiology & Research, School of Public Health, University of Cape Town; Sub-Saharan African Congenital Anomalies Network

**Keywords:** routine pregnancy surveillance, congenital disorders, neonatal surface examination

## Abstract

**Background:**

Lack of data on the burden and scope of congenital disorders (CD) in South Africa undermines resource allocation and limits the ability to detect signals from potentially teratogenic pregnancy exposures.

**Methods:**

We used routine electronic data in the Western Cape Pregnancy Exposure Registry (PER) to determine the overall and individual prevalence of CD identified on neonatal surface examination at birth in the Western Cape, South Africa,2016–2022. CD were confirmed by record review. The contribution of late (≤24 months) and antenatal diagnoses was assessed. We compared demographic and obstetric characteristics between women with/without pregnancies affected by CD.

**Results:**

Women with a viable pregnancy (>22 weeks gestation; birth weight≥500g) (n=32,494) were included. Of 1,106 potential CD identified, 56.1% were confirmed on folder review. When internal and minor CD were excluded the prevalence of major CD identified on surface examination at birth was 7.2/1000 births. When missed/late diagnoses on examination (16.8%) and ultrasound (6.8%) were included, the prevalence was 9.2/1000 births: 8.9/1000 livebirths and 21.5/1000 stillbirths. The PER did not detect 21.5% of major CD visible at birth. Older maternal age and diabetes mellitus were associated with an increased prevalence of CD. Women living with/without HIV (or the timing of antiretroviral therapy, before/after conception), hypertension or obesity did not significantly affect prevalence of CD.

**Conclusions:**

A surveillance system based on routine data successfully determined the prevalence of major CD identified on surface examination at birth at rates slightly higher than in equivalent studies. Overall rates, modelled at ~2%, are likely underestimated. Strengthening routine neonatal examination and clinical record-keeping could improve CD ascertainment.

## Introduction

Owing to improved treatment for HIV, malaria and measles; improved childhood vaccination; and greater access to quality ante- and perinatal care, congenital disorders (CD) account for an increasing proportion of neonatal and under-5 mortality, globally (1). In 2021 CD were the fifth leading cause of under-5 mortality and the third leading cause of neonatal deaths (2, 3). In addition, CD accounted for an estimated 7.4% of stillbirths (4).

Over 90% of the estimated 8 million children born with a serious CD annually are found in low- and middle-income countries (LMIC) where over 95% of CD-related deaths occur. This imbalance has been ascribed to poverty-related and infectious exposures that increase the risk of CD, nutritional deficiencies, toxic environmental exposures, fragmented and under-resourced health services, and the survival advantage against malaria for carriers of some single gene disorders (5).

Since 2006, with the improvement of obstetric and neonatal services in South Africa, most notably the introduction of antiretroviral therapy (ART) for the treatment of HIV and comprehensive vertical transmission prevention and early diagnosis of HIV, infant and child mortality have declined (6), and CD contribute a larger proportion to neonatal and childhood morbidity and mortality. In the Western Cape Province in South Africa fetal abnormalities were confirmed as the final cause of early neonatal death (eNND) in 13.5% of babies ≥500g and<1000g; and 24.3% of babies ≥1000g in the public health sector. In parts of Cape Town, fetal abnormalities were the leading cause of eNND in babies ≥1000g (33.3%) followed by hypoxia (29.5%), prematurity-related causes (15.9%), and infection (14.4%) (2017 – 2019) (7, 8).

There is a lack of empiric data on the rates of even common CD in South Africa. Since 2006, the South African Department of Health has been collecting CD data as part of a national passive surveillance system (9). A review of the methodology and data quality of this programme estimated that under-reporting of cases was over 98% (9). Modelling studies predict a birth prevalence of CD in South Africa of at least 27.6/1000 live births, with CD accounting for 5% of stillbirths, 57% for under-5 mortality and 36% of disabled survival at 5 years in the absence of interventions (10). The true scale and nature of CD in South Africa are unknown owing to unreliable reporting.

This lack of accurate data on the burden and scope of CD undermines appropriate resource allocation and the provision of quality health services for affected people. In addition, without data on baseline prevalence, it is not possible to detect deviations from the norm (signals) potentially due to medicine or other environmental exposures in pregnancy. To investigate associations between pregnancy exposures and CD, it is essential that rates in both live births and stillbirths are determined as well as rates of termination of pregnancy for fetal anomaly (TOPFA) (11). We determined prevalence rates of CD and associated factors over six years in a public health sector sentinel site-based pregnancy exposure registry in the Western Cape Province, South Africa (12).

## Methods

The Western Cape Pregnancy Exposure Registry (PER) was established in 2016 at two sentinel public sector obstetric sites in the Western Cape Province to provide ongoing surveillance of drug exposures in pregnancy and associations with pregnancy outcomes. Women attending antenatal care at primary care obstetric facilities (Midwife Obstetric Units; MOU) were enrolled and followed-up to pregnancy outcome regardless of the level of care (12). Exposure data were prospectively collected which limited recall bias, and the sample represents all women in the geographic areas served by the MOUs which avoided selection bias introduced by hospital-based studies. Surveillance was embedded in the services and routine clinical data were digitized using existing government-managed information systems. Data were collected from the clinical stationery used at the sites, namely the admission and delivery registers, hospital files and the Maternity Case Record (MCR). The MCR is a paper-based patient-held document that records all clinical obstetric information from the first antenatal visit to discharge post pregnancy outcome. It is retained at the site of delivery. The PER is situated within the Provincial Health Data Centre (PHDC), an information exchange that harmonises and consolidates multi-source electronic health data in the Western Cape (13). Over 30,000 women have been enrolled in the PER: approximately 5000 per year since 2016 from the Gugulethu MOU in Cape Town (urban) and approximately 7000 in total from the Worcester MOU between 2018–2019 (rural). Key obstetric and neonatal indicators have been validated against the aggregate data in the South African District Health Information System (12).

Examination of neonates and stillborn infants is standard of care in South Africa although often poorly performed and recorded in the case of stillbirths. The health service offers a dating obstetric ultrasound to all pregnant women, but coverage of any antenatal ultrasound was 38.4% in the Western Cape (2018 – 2019) owing to resource constraints (12). The Fetal Medicine Unit (FMU) at Groote Schuur Hospital in Cape Town is a tertiary referral unit to which patients with a suspected fetal abnormality or at risk of CD due to teratogen exposure, previous pregnancy affected by CD or advanced maternal age are referred. Women with fetuses affected by severe or life-threatening CD may be offered TOPFA. The FMU uses Astraia^™^ software (14) to generate and store antenatal data. Ultrasound findings, clinical data and management plans are recorded in real-time.

We defined a cohort of women in the PER with a pregnancy outcome after 22 weeks gestation or a baby of ≥500g between 01 September 2016 and 16 December 2022 (denominator). This ensured that terminations of pregnancy for CD after 22 weeks were included as well as being our definition of viability (4). Repeat pregnancies in the same woman and multiple pregnancies were included as the objective was to determine prevalence of major CD. Individual longitudinal data were available in the PHDC allowing for identification of CD diagnosed after birth, including internal CD. Potential CD were identified by neonatal surface examination as documented in the MCR and/or International Classification of Disease (ICD10)(15) coding in the electronic (PHDC) database and confirmed by folder review. PER data were linked per woman per pregnancy with the FMU database for the same period to determine the overlap between, and contribution of each database to the combined prevalence of CD.

Major CD were defined as abnormalities with medical, surgical, or cosmetic significance, i.e., any structural or functional anomaly with an impact on physical, intellectual, and social wellbeing (16, 17). The primary outcome was the prevalence per 1000 births of major CD that would be visible on surface examination of the neonate/stillbirth (i.e., excluding internal CD e.g., renal abnormalities or acyanotic congenital heart disease); and prevalence of CD with a delayed clinical presentation (acyanotic congenital heart disease, and some genetic disorders). Many specialist methods for diagnosis of CD (clinical specialists, diagnostic equipment) are not available in resource-constrained settings and most contemporary CD surveillance programmes in Africa use a limited surface examination of the neonate/stillborn infant by clinical staff (midwife or doctor) to identify CD (18–21). A validation study has determined that the limited surface examination can identify most visible external anomalies (major and minor), but not abnormalities inside the mouth (isolated cleft palate), most heart defects, peripheral vascular anomalies, and some internal anomalies, identifying 75% of expected malformations (22).

In this study, we present the prevalence of major CD that could be identified on external/surface examination of the neonate/stillbirth within 24 hours of birth. These included all major external anomalies; cyanotic (identified by central cyanosis) and critical congenital heart defects (hypoplastic left heart, pulmonary atresia, Tetralogy of Fallot, total anomalous pulmonary venous drainage, transposition of the great vessels, tricuspid atresia, truncus arteriosus, complex cardiac abnormalities, congenital heart block); congenital cataract (detected by absent red reflex); imperforate anus (failure to pass meconium); and atresias (detected by feeding difficulty/distended abdomen/bile-stained vomiting).

The rates of CD entered at birth in the PER have been presented to assess its effectiveness as a surveillance system based on the surface examination. In addition, the combined prevalence including late diagnoses in the PHDC (i.e., erroneously missed or not recorded at birth) and relevant CD in the FMU database have been included as a more accurate measure of ‘true prevalence’ of major CD that should be visible on external examination at birth.

Data were analyzed using STATA v.17 (StataCorp, Texas, USA). Descriptive statistics were used to describe the demographic and obstetric characteristics of women with pregnancies affected/not affected by CD. Continuous variables were described using medians with interquartile range and compared using the Mann Whitney U test. Categorical variables were presented as proportions and comparisons were made using the chi^2^ test or Fishers exact test as indicated. Prevalence rates were described per 1000 births. Agreement between the FMU database and the PER was assessed using Cohen’s kappa with 95% confidence intervals (CI). Kappa values were interpreted using the Landis and Koch categories (23): almost perfect (>0.80), substantial (0.61 – 0.80), moderate (0.41 – 0.60), fair (0.21 – 0.40), slight (0.00 – 0.20), and poor (<0.00).

Since we used routine health data not collected for research purposes, we applied the RECORD checklist (an extension of the STROBE criteria) (24).

## Ethical considerations

The study was reviewed and approved by the following Institutional Review Boards: the University of Cape Town Faculty of Health Sciences Human Research Ethics Committee (HREC REF: 791/2022; 541/2015 and 749/2015 [PER and PHDC]; 593/2010 and R019/2019 [FMU]) and the Western Cape Government Provincial Research Committee (WC_2016RP-286). All databases recorded routinely collected clinical data and a waiver of informed consent was granted. De-identified data were used for all analyses.

## Results

**Figure 1.**
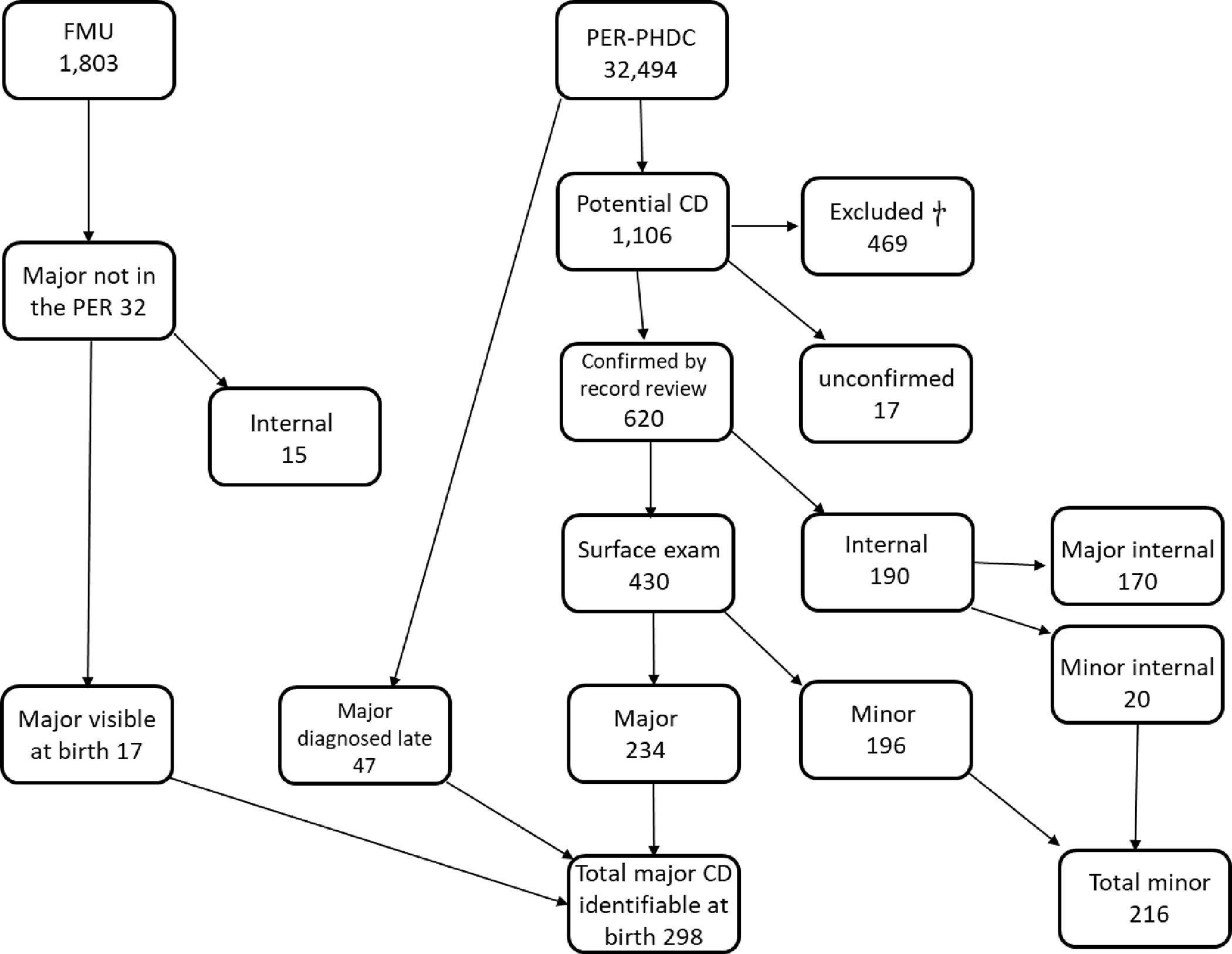
Major and Minor Congenital Disorders CD – congenital disorders; FMU – fetal medicine unit; PER – Pregnancy Exposure Registry; PHDC – Provincial Health Data Centre † see Supplementary Table 2

Over the study period, 32,494 pregnant women in the PER with infant gestational age ≥22 weeks or birth weight of ≥500g were included (Supplementary Figure 1). Of these, a live birth was recorded as the outcome in 31,751 (97.71%) and a stillbirth in 743 (2.29%). There were 207 neonatal deaths (0.65% of live births). Of the 1,106 potential CD identified in the PER, 620 (56.10%) were confirmed on folder review; 17 (1.54%) were unconfirmed owing to missing folders (Supplementary Table 1) and 469 (42.41%) were excluded giving a total prevalence rate for CD in the PER of 19.1/1000 births (including, major, minor and internal CD diagnosed up to 24 months old) (Figure 1). The main reasons for exclusion included a normal examination on folder review (44.14%), normal variation (19.19%), complications of prematurity (10.87%), illness unrelated to CD (10.45%) and positional deformities of the lower limbs (6.40%) (Supplementary Table 2).

When internal CD identified on antenatal ultrasound were excluded, 430 CD were identified by the neonatal surface examination at birth (prevalence 13.2/1000 births including major and minor CD). Minor CD comprised 34.84% of all CD (n=216; Supplementary Table 3), 180 (83.33%) of which were postaxial polydactyly Type B: giving a prevalence rate of 5.5/1000 live births. Minor disorders occurred in isolation (i.e., no infant with multiple minor anomalies) and the longitudinal data did not demonstrate prolonged follow-up.

The total major CD identified in the PER by the surface examination at birth was 234 giving a prevalence of 7.2/1000 births; 7.2/1000 live births (n=226) and 10.8/1000 stillbirths (n=8) (Figure1; Table 1).

We excluded internal anomalies diagnosed antenatally (which would not present at birth) or presenting >24 hours (renal anomalies, acyanotic cardiac lesions, biliary atresia) and those that only presented clinically after birth including some infants with fetal alcohol syndrome and genetic conditions, congenital sub-glottic stenosis and haemangiomas/lymphangiomas. Forty-seven infants had major CD that could have been identified on surface examination at birth but were diagnosed after day 1: Down Syndrome (n=13; median age at diagnosis 60 days [interquartile range (IQR) 45 – 156]), hypospadias (n=6; median age at diagnosis 51.5 days [IQR 45 – 80]), cervical meningocoele (n=1; age at diagnosis 90 days) and anorectal malformation (n=3; all diagnosed at 3 days). In addition, there were two infants with isolated cleft palate diagnosed at 5 and 120 days. Thus, almost 17% (16.8%) of major CD visible on surface examination at birth were not identified in the PER at birth (Figure 1; Table 1 and Supplementary Table 4.)

The FMU database included 1,803 individual pregnancies from the PER cohort. Thirty-two fetuses with major CD were present in the FMU database and not in the PER. Of these, 17 would have been identifiable on external surface examination at birth, 64.7% of which occurred in stillbirths (n=9) or neonatal deaths (n=2). Thus, the FMU identified an additional 6.77% of major CD that were visible by neonatal/stillbirth surface examination but which were not included in the PER at birth (Figure1, Table 1 and Supplementary Table 4). These included Trisomy 18 (n=1; stillbirth), chromosomal abnormalities not otherwise specified (NOS) (n=2; stillbirths), omphalocoele (n=1; stillbirth), skeletal dysplasia NOS (n=5; 3 stillbirth, 1 eNND, and 1 live birth), congenital syphilis (n=1; stillbirth), cleft lip and palate (n=1; live birth), and amniotic band (n=1; live birth) (Supplementary Table 4).

There was 72.53% (95% CI 60.60 – 85.45) agreement between the PER and FMU datasets. Inter-database reliability by Cohen’s kappa was 0.41 (95% CI 0.25 – 0.56) indicating moderate agreement (23) (data not shown).

When infants with a delayed diagnosis (n=47) and those in the FMU dataset only (n=17) were included, the number of major CD that could be identified by surface examination at birth was 298, equating to a 0.92% prevalence of major CD 8.9/1000 live births and 21.5/1000 stillbirths were affected by CD (Table 1). The PER missed a total of 21.5% major CD visible at birth.

The prevalence of major CD visible at birth varied between 7.9/1000 and 10.8/1000 over the study period with and overall prevalence 9.2/1000. There was an increase in proportion of the CD missed on external examination at birth and diagnosed late in 2020, coinciding with the first year of the SARS-CoV-2 pandemic (26.32% CD were missed in 2020 versus 16.39% in 2019 and 8.33% in 2021). There were no obvious patterns in terms of the types of CDs by year.

The most common category of CD was congenital heart disease not associated with a sequence or chromosomal disorders (n=72; prevalence of 2.3/1000 births up to 24 months old) (Table 2 and Supplementary Table 5). We determined that cyanotic and critical heart disease should be possible to diagnose by external neonatal examination at birth and these had a prevalence of 0.8/1000 births. The median age of diagnosis of all cardiac lesions was 6.5 days (IQR 0 – 87).

The prevalence of chromosomal abnormalities was 1.9/1000 births, which were mostly Down Syndrome (1.4/1000 births) and Trisomy 18 (0.25/1000 births) (Table 2). Of the 43 live born infants with Down Syndrome, 24 were diagnosed with congenital heart disease, most commonly atrioventricular septal defect.

Other common CD visible on external neonatal examination at birth were genito-urinary anomalies (1.8/1000 births) and musculo-skeletal anomalies (1.2/1000 births). Prevalence of talipes was 0.34/1000 births. Neural tube defects (NTD) were present in 0.52/1000 births (myelomeningocoele [n=9], spina bifida NOS [n=3], cervical meningocoele [n=1], spina bifida occulta [n=2, one of which required surgery], anencephaly [n=1] and frontal encephalocoele [n=1]). 1.3/1000 births were affected by CD associated with confirmed teratogen exposure i.e., positive titers for syphilis or CMV, confirmed antenatal alcohol, methamphetamine or sodium valproate exposure with a typical clinical phenotype, and diabetes mellitus (Table 2).

Women with pregnancies affected by CD were older than those unaffected (median age 28.8 years [IQR 23.5 – 35.6] versus 27.2 [IQR 22.7 – 32.3]; p=0.015) and a higher proportion had diabetes mellitus (6.4% versus 3.1%; p=0.001) (Table 3). There were no statistically significant differences in terms of HIV infection, hypertension, and obesity between the two groups. Rates of overweight/obesity (as determined by body mass index [BMI] and mid-upper arm circumference [MUAC]) were high in both. Among women living with HIV, the timing of ART initiation (i.e., preconception versus during pregnancy) was not different between women with pregnancies affected by CD and not. There were no differences in exposure to alcohol, cigarette smoking and recreational drugs between groups although the reported numbers of these exposures in the study population were low. Women with pregnancies affected by CD were more likely to have had an obstetric ultrasound and to have delivered in a specialist hospital (26.9% versus 6.0% Level 3; p<0.0001). In all CD-affected pregnancies, the proportions with stillbirth and neonatal death were more common than in those not affected by CD (i5% versus 2.3%; p<0.0001 and 9.3% versus 0.6%; p<0.0001, respectively) as were prematurity and low birth weight (Table 3). During 2018 – 2019 when two sites contributed data to the PER, it was not possible to differentiate the prevalence between the urban (Gugulethu MOU) the rural (Worcester MOU) sites (0.75% versus 0.66%; p=0.508) (Table 3.)

## Discussion

The PER, by digitizing routine clinical data, is an effective system to monitor prevalence of major CD visible on external neonatal/stillbirth examination at birth (i.e., 7.2/1000 births.) If late diagnoses and those in the FMU database were included the overall prevalence of major CD identifiable on surface examination at birth was 9.2/1000 births, with rates higher in stillbirths (21.5/1000) versus live births (8.9/1000)(Table 1). This difference is not unexpected as CD are more common and often more severe in stillbirths (23). These rates are slightly higher than those found in similar studies that depended on surface examination at birth for diagnosis (photographs were validated by external review where possible): 6.0/1000 in the Tsepamo study in Botswana(26), 8.0/1000 in Eswatini (20), 3.6/1000 in Malawi (19), and 5.0/1000 in KwaZulu-Natal Province in South Africa (27). A systematic review of CD in Africa calculated the pooled prevalence as 23.5/1000 (16.6/1000 in South Africa) (28). However, this was not restricted to major anomalies identified on surface examination at birth and studies on CD in sub-Saharan Africa are dominated by only four (out of 49) countries (Nigeria, Ethiopia, South Africa and Uganda), more likely a reflection of medical and research resources, rather than true prevalence (29). Our prevalence is also lower than the modelled birth prevalence of CD in South Africa, 27.6/1000 live births, although this figure includes internal anomalies and those presenting clinically after birth (10). Globally, the prevalence of major CD is estimated to be approximately 2 –3% of births (e.g., 3.0/1000 in China (30); 2.7/1000 in the EUROCAT database (31); 2.23/1000 in the UK National Congenital Anomaly and Rare Disease Registration Service (NCARDRS) (32); and 2.8/1000 in the USA (33)). If we assume that prevalence of major CD in South Africa is similar (~2–3%), then there is overall under-ascertainment of CD in the PER. However, ascertainment by surface examination at birth in comparison with similar studies is reassuring.

The embedding of the PER within the PHDC has the added advantage of longitudinal follow-up of infants and children beyond birth allowing for identification of late/missed diagnoses that should have been made at birth. However, 42% of CD identified in the electronic data were excluded on folder review. It has been observed that a wide range (up to 24%) of notable physical features in neonates (normal variations, position deformities, birth marks, prematurity-related features) do not meet the definition of CD (34). Post-hoc reviews of neonatal examinations in Botswana confirmed CD in 70% of those initially identified; only 30% of these were major CD and 40% of them were diagnosed late (i.e., after birth) (35). In addition, trauma, infection and other neonatal illnesses and cerebral palsy related to peri/post-natal insults were miscoded with CD ICD10 codes in the PHDC (Supplementary Table 2). An assessment of the validity of diagnosis and procedure codes for NTD in electronic healthcare data concluded that in many cases they did not accurately identify CD (positive predictive value 41%) (36). The misclassification in the PER-PHDC dataset would overestimate the prevalence of CD without the more resource-intensive folder review. The UK NCARDRS operates a multisource electronic database similar to the PHDC; however, the data for each clinical record are processed and combined by trained registration officers to ensure accurate classification (32). Such resources are currently unavailable in the South African setting.

The PER missed 21% of major CD that were visible on external examination at birth (16.8% missed on physical examination and 6.8% of mainly stillbirths and eNND identified in the FMU antenatal ultrasound database only). Most of those missed were in 2020, the first year of the SARS-CoV-2 pandemic in which lockdown directives and clinical pressures disrupted the health services with direction of resources to emergency care. Notwithstanding this disruption, improved and more frequent training in the neonatal surface examination, especially of stillbirths, and good clinical record-keeping could improve these rates. Examination of stillbirths has been noted to be low in other LMIC settings with implications for determination of CD prevalence (37). New technologies, such as the Global Birth Defects Description and Coding App could assist with diagnosis of CD in resource-constrained settings (38) as could the routine use of pre-discharge pulse oximetry for all newborns (39).

Many contemporary CD surveillance programmes in sub-Saharan Africa were established to monitor birth outcomes in women exposed to ART regimens for prevention and treatment of HIV (18–21, 27, 40) and anti-malarial agents for the prevention and treatment of malaria (41) in pregnancy. This gained urgency in 2018 when the Tsepamo study in Botswana identified a safety signal of increased NTD in women living with HIV who had been exposed to the integrase inhibitor, dolutegravir around the time of conception (21). Expansion of the Tsepamo sample resulted in a decrease in the effect size (26); however, the episode highlighted the importance of routine pregnancy surveillance systems, especially in regions in which pregnant women and women of child-bearing age are exposed to potentially teratogenic agents at scale. Subsequent studies have failed to demonstrate an increased risk of NTD in women receiving dolutegravir in pregnancy (19, 20). These analyses do not assess teratogenicity and or exposure to individual therapeutic agents which are the subject of future work. We did not observe an increased prevalence of CD in women living with HIV versus those who were not, nor in those who were taking ART around the time of conception versus those who started/restarted treatment during pregnancy. The prevalence of NTD was 0.52/1000 births, lower than in similar studies in which prevalence ranges from 0.57/1000 – 0.98/1000 births (18–20, 30, 42, 43), and lower than the modelled estimates (0.98/1000 = 1.2/1000 births) (10, 31), perhaps reflecting under-ascertainment.

Ideally, when assessing the potential impact of pregnancy exposures (whether teratogenic or protective e.g., folate supplementation) *all* outcomes should be evaluated. This is challenging in many settings, including the Western Cape, where early elective terminations of pregnancy and miscarriages are often not included.

### Strengths and Limitations

The prospective design of the PER is a strength of the programme, allowing for the definition of a denominator group representing the geographic area supported by the MOU. Most women in the Western Cape deliver at a health care facility or attend a facility with their infants shortly after delivery making this an appropriate choice. Enrolment at a primary health care site reduces the selection bias of hospital-based studies which may select for higher-risk pregnancies. Approximately 50% of women who attend antenatal care at primary obstetric sites will deliver at these sites, with the remainder being referred to hospital ante- or perinatally (12).

While we do not have photographs of suspected CD for expert review and confirmation of diagnosis as in similar studies, the majority of live infants with suspected CD in the Western Cape were referred for specialist care at Level 3 hospitals (with neonatal, paediatric medical and surgical specialist care) where a diagnosis was confirmed/excluded. The Western Cape is one of the two out of a total of nine provinces in South Africa with public sector specialist clinical genetic services.

The main limitation of the study is due to our dependence on routine clinical data recorded by the attending clinicians and facility clerical staff. We were unable to account for missing and incorrect data so misclassification could have occurred. As noted, the ICD10 coding in the electronic PHDC dataset was incorrect in some cases. We attempted to reduce this risk with detailed folder review of suspected CD and in 17 cases medical records were unavailable. Specific detail was lacking for some CD e.g., categories of hypospadias, specific chromosomal diagnoses, and there was a selection bias towards CD in live births as opposed to stillborn infants.

We only included women with a viable pregnancy defined as >22 weeks gestation or a baby birth weight of ≥500g. In this cohort there were no terminations of pregnancy for fetal anomaly using this definition. We did not include terminations of pregnancy before 22 weeks gestation. In South Africa, termination of pregnancy is available on request up to 12 weeks gestation and under certain conditions up to 20 weeks gestation. The PER dataset did not support a more detailed assessment. Termination of pregnancies potentially affected by CD before 22 weeks gestation were not included, and this may have contributed to the underestimation of the prevalence of CD in the cohort.

BMI was calculated from the first weight measurement and is not an ideal calculation during pregnancy; however, we did not have serial weight data to determine gestational weight gain. Of note, differences in MUAC, which can be used as an indirect measure of overweight/obesity during pregnancy were not significant either in women with pregnancies affected by CD or not (44).

## Conclusions

The PER, a routine pregnancy surveillance system integrated into existing data management services in the public health sector was able to consistently determine overall and individual prevalence rates for common CD visible on neonatal surface examination at birth comparable to similar programmes in sub-Saharan Africa. Integration with the electronic information exchange (PHDC) strengthened the system allowing for inclusion of CD identified after birth (i.e., > 24 hours old). There is immense value in combining data from multiple sources and the routine integration of the relevant FMU data would further improve CD ascertainment. However, review of suspected CD cases is still required. Some of the limitations of the PER in its current form can be addressed by additional and ongoing training in clinical examination and clinical record-keeping and an appreciation of the value of formal examination of stillborn infants. These services should be standard of care. The PER provides a platform which can describe prevalence and determine associations and changes related to pregnancy exposures.

Valid data are required to determine the burden of and risks for CD and to evaluate the outcome of interventions. Resource constraints limit the application of active surveillance nationally. However, sentinel rates can support modelling, planning, allocation resources as well as identify clusters of cases (signals) that may indicate dangerous exposures in pregnancy (31).

## Supplementary Material

Supplementary info

2

## Figures and Tables

**Table 1.** Prevalence of Congenital Disorders in the Pregnancy Exposure Registry and Fetal Medicine Unit Datasets

	n (%)	Prevalence			Prevalence/1000 births		

		Total n = 32,494	Live births n=31,751	Still births n=743	Total	Live births	Still births

**Total CD in the PER**	620	1.91%			19.1/1000		
Internal ^†^	190 (30.65)						
Diagnosed on external surface examination at birth	430 (69.35%)	1.32%			13.2/1000		

**Minor CD**	216	0.66%			6.6/1000		
Diagnosed on external surface examination at birth	196 (90.74)	0.60%	0.60%			6.0/1000	
Post-axial polydactyly Type B	180 (83.33)	0.55%	0.55%			5.5/1000	
Minor internal anomalies	20 (9.26)	0.06%	0.06%			0.6/1000	

**Major CD**							
Total major CD in the PER	404	1.24%			12.4/1000		
Median age at diagnosis (days) (IQR)	0 (0 – 63.5)						
**Diagnosed on external surface examination at birth**	**234**	**0.72%**	0.72%	1.08%	7.2/1000	7.2/1000	10.8/1000

Diagnosis missed on external surface examination at birth	47 (16.8) ^‡^	0.14%				1.4/1000	

Diagnosis in FMU database only (but visible on external surface examination at birth)	17 (6.77) ^‡^	0.05%				0.5/1000	

**All inclusive major CD visible on external surface examination at birth**	**298**	**0.92%**	**0.89%**	**2.15%**	9.2/1000	8.9/1000	21.5/1000

All major CD in PER-PHDC-FMU							
(external, internal PER, late PHDC, external and internal FMU)	558	1.72%			17.2/1000		

CD – congenital disorder; FMU – Fetal Medicine Unit; IQR – interquartile range; PHDC – Provincial Health Data Centre; PER – Pregnancy Exposure Registry

† diagnosed by antenatal ultrasound

‡ percentage CD diagnoses missed of 281 (234+47) and 251 (234+17), respectively

**Table 2.** Prevalence of specific congenital disorders visible on external neonatal examination at birth

	PER				Inclusive †			

	Prevalence/1000	Total	Live Birth (NND)	Stillbirth	Prevalence/1000	Total	Live birth (NND)	Stillbirth

Chromosomal	1.3/1000	43	41	2	1.9/1000	62	49	3
Trisomy 21	0.95/1000	31	30	1	1.4/1000	44	43	1
Trisomy 18	0.21/1000	7	6 (6)	1	0.25/1000	8	6 (6)	2
Other chromosomal^‡^	0.15/1000	5	5 (1)	0	0.3/1000	10	8 (1)	2

Genetic	0.31/1000	10	9 (3)	1	0.043	14	13	1

CNS	0.68/1000	22	21 (4)	1	0.86/1000	28	26	2
Neural tube defects	049/1000	16	15 (3)	1	0.52/1000	17	16 (3)	1
Other CNS^‡^	0.18/1000	6	6 (1)	0	0.33/1000	11	10 (1)	1

Orofacial clefts (isolated)	0.40/1000	13	12	1	0. 43/1000	14	13	1

Gastrointestinal tract	0.46/1000	15	15 (2)	0	0.64/1000	21	20 (2)	1
Abdominal wall defects	0.21/1000	7	7 (1)	0	0.27/1000	9	8 (1)	1
Atresias	0.25/1000	8	8 (1)	0	0.31/1000	10	10 (1)	0
Other GIT^‡^	0	0	0	0	0.06/1000	2	2	0

Genito-urinary	0.68/1000	22	22	0	1.8/1000	58	58	0
Hypospadias	0.40/1000	13	13	0	0.58/1000	19	19	0
Undescended testes^§^	0.06/1000	2	2	0	0.92/1000	30	30	0
Ambiguous genitalia	0.22/1000	7	7	0	0.28/1000	9	9	0

Musculo-skeletal	0.89/1000	29	29 (2)	0	1.10/1000	35	32	3
Talipes equinovarus	0.34/1000	11	11	0	0.34/1000	11	11	0
Skeletal dysplasia	0.09/1000	3	3 (3)	0	0.18/1000	6	5 (4)	2
Other musc-skeletal^‡^	0.46/1000	15	15 (1)	0	0.55/1000	18	17 (1)	1

Teratogen exposure	0.34/1000	11	11	0	0.36/1000	12	11	1
Fetal alcohol syndrome	0.15/1000	5	5	0	0.15/1000	5	5	0
Valproate embryopathy	0.03/1000	1	1	0	0.03/1000	1	1	0
Congenital CMV syndrome	0.12/1000	4	4	0	0.12/1000	4	4	0
Congenital syphilis	0.03/1000	1	1	0	0.06/1000	2	1	1
Diabetes mellitus	0.40/1000	13	12 (2)	1	0.58/1000	19	16 (4)	3

Other	1.5/1000	49	46	3	1.7/1000	56	53	3
Amniotic band syndrome	0.12/1000	4	4	0	0.15/1000	5	5	0
Aglossia	0.03/1000	1	1	0	0.03/1000	1	1	0
Dysmorphic NOS	0.22/1000	7	7	0	0.22/1000	7	7	0
Lymphangioma face	0.03/1000	1	1	0	0.03/1000	1	1	0
Portwine stain face	0.06/1000	2	2	0	0.06/1000	2	2	0
Multiple	0.58/1000	19	16	3	0.58/1000	19	16	3
Third branchial arch remnant	0.03/1000	1	1	0	0.03/1000	1	1	0
VACTERL	0.09/1000	3	3	0	0.09/1000	3	3	0
Congenital cataract	0.03/1000	1	1	0	0.15/1000	5	5	0
Congenital diaphragmatic	0.09/1000	3	3	0	0.12/1000	4	4	0
hernia								0
Congenital sub-glottic	0.06/1000	2	2	0	0.06/1000	2	2	0
stenosis	0.03/1000	1	1	0	0.03/1000	1	1	0
Dermoid cyst head	0.03/1000	1	1	0	0.03/1000	1	1	0
Congenital anomaly NOS	0.03/1000	1	1	0	0.03/1000	1	1	0
Laryngeal cleft	0.03/1000	1	1	0	0.03/1000	1	1	0
Occulo-auriculo-vertebral								0
Syndrome	0.03/1000	1	1	0	0.03/1000	1	1	0
Pulmonary hypoplasia	0	0	0	0	0.03/1000	1	1	0
Capillary malformation NOS	0	0	0	0	0.03/1000	1	1	0

Cardiac	0. 34/1000	11	11	0	0.77/1000	25	25	0
Tetralogy of Fallot	0.09/1000	3 (2)	3 (2)	0	0.31/1000	10 (2)	10 (2)	0
Total anomalous pulmonary	0.06/1000	2	2	0	0.12/1000	3	3	0
venous drainage								0
Complex cardiac	0.09/1000	3 (3)	3 (3)	0	0.15/1000	5 (3)	5 (3)	0
Cardiac NOS	0.03/1000	1	1	0	0.12/1000	4 (1)	4 (1)	0
Hypoplastic left heart	0.03/1000	1	1	0	0.03/1000	1	1	0
Coarctation of the aorta	0	0	0	0	0.03/1000	1	1	0
Congenital heart block	0.03/1000	1	1	0	0.03/1000	1	1	0

Acyanotic cardiac lesions	0.28/1000	9	9	0				
Atrial septal defect								
Ventricular septal defect	0.06/1000	2	2	0				
Atrioventricular Septal Defect	0.09/1000	3	3	0				
anomalous left coronary	0.06/1000	2	2	0	not diagnosed at birth			
artery from the pulmonary	0.03/1000	1	1	0				
artery								
Pulmonary stenosis	0.03/1000	1	1	0				

Total cardiac diagnosed at birth	0.62/1000	20	20	0				

Total	7.2/1000	234			9.2/1000	298	8.9/1000	21.5/1000

CAH – congenital adrenal hyperplasia; CMV – Cytomegalovirus; CNS – Central Nervous System; GIT – Gastrointestinal Tract; NND – Neonatal Death; NOS – not otherwise specified; VACTERL – vertebral defects, anal atresia, cardiac defects, trachea-oesophageal fistula, renal anomalies, limb abnormalities

† Inclusive includes the 47 and 17 CD visible on external neonatal examination at birth diagnosed late and in the FMU dataset only, respectively

‡ Other chromosomal abnormalities: chromosomal abnormality NOS (1), Di George Anomaly (1), Prader Willi Syndrome (1), Turner Syndrome (1), Trisomy 13 (1)

Genetic disoders: Hirschsprung’s Disease (1), Mabry Syndrome (1), Neurofibromatosis (1), Saethre-Chotzen Syndrome (1), Sturge Weber Syndrome (1), Thanatropic dysplasia (1), X-linked Myotubular Myopathy (1), Achondroplasia (3)

Neural tube defects: anencephaly (1), myelomeningocoele (9), spina bifida NOS (3), frontal encephalocoele (1), cervical mengingocoele (1), spina bifida occulta (2)

Other CNS abnormalities: craniosynestosis (1), holoprosencephaly (1), hydranencephaly (1), hydrocephalus (1), macrocephaly (2), microcephaly (2), ventriculomegaly (2)

Orofacial clefts: isolated cleft lip (4), cleft lip and palate (3), isolated cleft palate (7), orofacial cleft NOS (2)

Abdominal wall defects: omphalocoele (3); gastroschisis (4; 1 with intestinal atresia), Prune Belly Syndrome (1)

Atresia: anorectal malformation (5), intestinal atresia (3), oesophageal atresia (3);

Other GIT: malrotation bowel (1) GIT NOS (1)

Ambiguous genitalia includes one confirmed CAH

Other musculo-skeletal abnormalities: Syndactyly (8), oligodactyly (1), pre-axial polydactyly (1), absent arm (1), absent halluces (1), congenital abnormality of the hand NOS (1), juvenile idiopathic scoliosis (1); congenital dislocation of the hip (1); short femur (1); congenital short stature (1)

§ Undescended testes at term; required surgical repair

**Table 3.** Cohort Characteristics

Characteristic	Total n=32,494	Women not affected by CD n=32,196 (99.08%)	Women with pregnancy affected by CD n=298 (0.92%)	p-value

Age (years) median (IQR)	27.2 (22.7 – 32.3)	27.2 (22.7 – 32.3)	28.8 (23.5 – 35.6)	0.015

Primigravida n (%)	9,320 (28.7%)	9,246 (28.7)	74 (24.8)	0.14

Site (limited to deliveries 2018–2019) n=15,619				
Gugulethu MOU	9,284 (59.4)	9,191 (99.00)	93 (1.00)	0.14
Worcester MOU	6,335 (40.6)	6,286 (99.23)	49 (0.77)	

Attendance antenatal care	1,058 (3.3)	1,050 (3.3)	8 (2,7)	0.577

Gestational age at booking (weeks) median (IQR)	18.3 (12.3–24.3)	18.3 (12.3 – 24.7)	18.6 (12.7 – 24.3)	0.859

Antenatal ultrasound n (%)	9,997 (30.8)	9.852 (30.6)	145 (48.7)	<0.001

Delivery location n (%)				<0.001
MOU	13,559 (41.7)	13,493 (41.9)	66 (22.2)	
Level 1	960 (3.0)	943 (2.9)	17 (5.7)	
Level 2	15,269 (47.0)	15,142 (47.0)	127 (42.6)	
Level 3	2,010 (6.2)	1,930 (6.0)	80 (26.9)	
Unknown	696 (2.1)	688 (2.1)	8 (2.6)	

Living with HIV n (%)	10,302 (31.7)	10,197 (31.7)	105 (35.2)	0.166
Unknown	657 (2.0)	648 (2.0)	9 (3.0)	
In WLHIV n = 10,302 n (%)				
ART preconception	7,860 (76.3)	7,773 (76.2)	87 (82.9)	0.216
ART start/restart during pregnancy	2,346 (22.8)	2,328 (22.8)	18 (17.1)	
ART timing unknown	96 (0.9)	96 (0.9)	0	

Diabetes n (%)	1,007 (3.1)	988 (3.1)	19 (6.4)	0.001
Pre-existing diabetes	367 (1.1)	360 (1.1)	7 (2.4)	0.045
Gestational diabetes	640 (2.0)	628 (2.0)	12 (4.0)	0.01

Hypertension n (%)	1,503 (4.6)	1,448 (4.6)	15 (5.0)	0.736
Pre-existing hypertension	1,282 (4.0)	1,271 (4.0)	11 (3.7)	0.821
Pregnancy-induced hypertension	221 (0.7)	217 (0.7)	4 (1.3)	0.162

Maternal Weight n (%)				0.324
Normal (BMI<25)	6,157 (19.0)	6,101 (19.0)	56 (18.8)	
Overweight (BMI 25–29.9)	6,780 (20.1)	6,730 (20.9)	50 (16.8)	
Obese (BMI>30)	12,471 (38.4)	12,346 (38.4)	125 (42.0)	
Unknown	7,086 (21.8)	7,019 (21.8)	67 (22.5)	
MUAC (cm) Median (IQR) (n = 27,340)	29.5 (26–33)	29.5 (26–33)	30 (36.7–34)	0.195

Smoking n (%)				0.171
Yes	2,360 (7.3)	2,333 (7.3)	27 (9.1)	
No	28,511 (87.7)	28,260 (87.8)	251 (84.2)	
Unknown	1,623 (5.0)	1,603 (5.0)	20 (6.7)	

Alcohol n (%)				0.22
Yes	1,571 (4.8)	1,551 (4.8)	20 (6.7)	
No	28,973 (89.2)	28,716 (89.2)	257 (86.2)	
Unknown	1,950 (6.0)	1,929 (6.0)	21 (7.1)	

Recreational drugs n (%)				0.111
Yes	70 (0.2)	68 (0.2)	2 (0.7)	
No	30,453 (93.7)	30,180 (93.7)	273 (91.6)	
Unknown	1,971 (6.1)	1,948 (6.1)	23 (7.7)	

Previous adverse birth outcome				
Yes	6,203 (19.1)	6,130 (19.0)	73 (24.5)	<0.0001
No	25,644 (78.9)	25,430 (79.0)	214 (71.8)	
Unknown	647 (2.0)	636 (2.0)	11 (3.7)	

Gestational age at delivery	39 (38 – 40)	39 (38 – 40)	38 (36 – 40)	<0.0001
Prematurity n(%)	7.944 (24.1)	7,828 (24.30)	116 (38.9)	<0.0001

Infant sex n (%)				
Female	14,810(45.6)	14,681 (15.6)	129 (43.3)	0.001
Male	15,122 (46.5)	14,989 (46.6)	133 (44.6)	
Indeterminate	8 (0.02)	6 (0.02)	2 (0.67)	
Not recorded	2,554 (7.9)	2,520 (7.8)	34 (11.4)	

Pregnancy outcome n (%)				<0.0001
Live birth	31,751 (97.7)	31,470 (97.8)	281 (94.3)	
Stillbirth	743 (2.3)	726 (2.3)	15 (5.0)	
Elective TOPFA			2 (0.7)	

Multiple pregnancy n (%)	779 (2.4)	772 (2.4)	7 (2.4)	0.577

Birth weight (g) median (IQR)	3100 (2730 –3430)	3100 (2740 – 3430)	2835 (2180–3280)	<0.0001
LBW n (%)	4,923 (15.2)	4,823 (15.0)	100 (33.6)	<0.0001

Length (cm) median (IQR)	50 (48 – 52)	50 (48 – 52)	48 (44 – 51)	<0.0001

Head (cm) median (IQR)	34 (33 – 35)	34 (33 – 35)	34 (32 – 35.5)	0.041

Neonatal death (of 31,751 livebirths) n (%)	207 (0.7)	181 (0.6)	26 (9.3)	<0.0001

ART - Antiretroviral Therapy; BMI – Body Mass Index; CD – Congenital Disorder; HIV – Human Immune Deficiency Virus; IQR – Interquartile Range; LBW – low birthweight (<2500g); MOU – Midwife Obstetric Unit; MUAC – Mid-upper Arm Circumference; TOPFA – Termination of Pregnancy for Fetal Anomaly; WLHIV – Women living with HIV

## Data Availability

Data are under the custodianship of the Western Cape Government Department of Health & Wellness and are not freely available. Application can be made to phdc.pgwc@westerncape.gov.za.
