# Supplementary material for: Population-based prevalence of congenital defects in a routine sentinel-site based surveillance system in the Western Cape, South Africa
Source: Birth Defects Res. Author manuscript; Available in PMC 2025 Jan 15. (PMC11733423; doi:10.1002/bdr2.2388)
Supplement: Supplementary info [file NIHMS2043832-supplement-Supplementary_info.docx]

**Supplementary Information**

**Supplementary Table 1, Unconfirmed Congenital Disorders identified by the Provincial Health Data Centre**

| **Diagnosis** | **Number (%)** n=17 |
| --- | --- |
| Dysmorphic NOS | 3 (17,65%) |
| Developmental delay NOS | 1 (5,88%) |
| Tetralogy of Fallot | 1 (5,88%) |
| Common arterial trunk (eNND) | 1 (5,88%) |
| Cardiac NOS (eNND) | 1 (5,88%) |
| Congenital malformation of the feet | 1 (5,88%) |
| Talipes | 1 (5,88%) |
| Congenital corneal opacity | 1 (5,88%) |
| Corneal pigmentation | 1 (5,88%) |
| Congenital ptosis | 1 (5,88%) |
| Ehlers-Danlos Syndrome | 1 (5,88%) |
| Duplicate ureter | 1 (5,88%) |
| Neck mass | 1 (5,88%) |
| No data | 1 (5,88%) |
| Skeletal dysplasia (eNND) | 1 (5,88%) |

eNND – early Neonatal Death (<7 days); NOS- not otherwise specified

**Supplementary Table 2, Reasons for Exclusion as a Congenital Disorder after Folder Review**

| **Category** | **Diagnosis** | **Number (%)** |
| --- | --- | --- |
| Normal examination (n=27; 44,14%) | None | 27 (100%) |
| Normal variation (n=90; 19,19%) | Undescended testes at term – no surgery  Ankyloglossia  “Soft dysmorphism”, no follow-up  Low set ears  Naevus  Benign cardiac murmur  Facial asymmetry  Macrosomia  Pilonidal cyst  Sacral dimple  Umbilical hernia  Café au lait spots x 2  Big eyes  Big head  Caput  Elongated head  Epicanthic folds  Moles on ear  Natal teeth  Sandal gap  Short-sighted  Skin tag  Small eyes  Sublingual cyst | 25 (27,78%)  14 (15,56%)  9 (10,00%)  8 (8,89%)  6 (6,67%)  5 (5,55%)  2 (2,22%)  2 (2,22%)  2 (2,22%)  2 (2,22%)  2 (2,22%)  1 (1,11%)  1 (1,11%)  1 (1,11%)  1 (1,11%)  1 (1,11%)  1 (1,11%)  1 (1,11%)  1 (1,11%)  1 (1,11%)  1 (1,11%)  1 (1,11%)  1 (1,11%)  1 (1,11%) |
| Complications of prematurity (n=51; 10,87%)  Gestational age < 37 weeks | Patent Ductus Arteriosus  Undescended testes  Inguinal hernia  Necrotizing enterocolitis  Immature genitalia  Complications of prematurity NOS | 32 (62,75%)  9 (17,65%)  6 (11,76%)  2 (3,92%)  1 (1,96%)  1 (1,96%) |
| Illness (n=49; 10,45%) | Prematurity  Respiratory distress syndrome  Blocked lacrimal duct  Fusion of labia  Early neonatal death  Post-procedural sub-glottic stenosis  Acquired sub-glottic stenosis  Acquired oesophageal stenosis  Acquired cerebral cyst  Asthma  Born flat  Cerebral cyst secondary to bleed  Croup  Developmental disorder  Encephalopathy  Distended abdomen  Floppy and flat  Gastroesophageal reflux disease  Hypoxic-ischemic encephalopathy  Hydrocephalus due to post-natal insult  Neonatal hepatitis syndrome  Neonatal jaundice  Pallor  Rash NOS  Sleep apnea | 16 (32,65%)  4 (8,16%)  3 (6,12%)  3 (6,12%)  2 (4,08%)  2 (4,08%)  1 (2,04%)  1 (2,04%)  1 (2,04%)  1 (2,04%)  1 (2,04%)  1 (2,04%)  1 (2,04%)  1 (2,04%)  1 (2,04%)  1 (2,04%)  1 (2,04%)  1 (2,04%)  1 (2,04%)  1 (2,04%)  1 (2,04%)  1 (2,04%)  1 (2,04%)  1 (2,04%)  1 (2,04%) |
| Positional deformity of the lower limbs (n=30; 6,4%) | Positional club foot  Positional foot  Genu recurvatum  Concern about hip – not confirmed  Bowing of the long bone  Congenital malformation of the hip – not confirmed  Congenital malformation of the knee – not confirmed  Hip clicks  Hyperflexed legs  Pes planus  Positional knee  Positional leg | 13 (43,33%)  5 (16,67%)  3 (10,00%)  1 (3,33%)  1 (3,33%)  1 (3,33%)  1 (3,33%)  1 (3,33%)  1 (3,33%)  1 (3,33%)  1 (3,33%)  1 (3,33%) |
| Trauma (n=15; 3,2%) | Skull fracture  Head injury  Shoulder dystocia  Cranial nerve VII palsy  Erb’s palsy  Cephalohaematoma  Humerus fracture  Ankle injury  Hip injury  Laceration to eyelid | 4 (26,67%)  2 (13,33%)  2 (13,33%)  1 (6,67%)  1 (6,67%)  1 (6,67%)  1 (6,67%)  1 (6,67%)  1 (6,67%)  1 (6,67%) |
| Infection (n=11; 2,35%) | Tuberculosis disease  Syphilis – no congenital malformations  Cerebral cyst due to infection  Bacterial meningitis  Cellulitis of the scrotum  Deafness due to meningitis  Hydrocephalus due to TB meningitis  Hydrocephalus due to GBS meningitis | 3 (27,27%)  2 (18,18%)  1 (9,09%)  1 (9,09%)  1 (9,09%)  1 (9,09%)  1 (9,09%)  1 (9,09%) |
| Cerebral palsy (n=10; 2,13%) | Cerebral palsy post infection  Cerebral palsy due to perinatal insult  Cerebral palsy NOS  Macrocephaly due to postnatal insult  Hypoxic brain injury at 19m old | 4 (40,00%)  2 (20,00%)  2 (20,00%)  1 (10,00%)  1 (10,00%) |
| Miscode (n=4; 0,85%) | Brain abnormality  Eclampsia  Spina bifida  NOS | 1 (25,00%)  1 (25,00%)  1 (25,00%)  1 (25,00%) |
| Lymph node (n=1; 0,21%) | None |  |
| Developmental delay NOS (n=1; 0,21%) | None |  |

GBS- group B streptococcus infection; NOS – not otherwise specified; TB – tuberculosis disease

**Supplementary Table 3, Minor Congenital Disorders in the Pregnancy Exposure Registry (internal & external)**

| **Category** | **Diagnosis** | **Number (%) n= 216** |
| --- | --- | --- |
| Hands & feet (n=183; 84,72%) | Post-axial polydactyly type B^ⴕ^  Campylodactyly  Oligodactyly  Hypoplastic 5^th^ toe | 180 (83,33)  1 (0,46)  1 (0,46)  1 (0,46) |
| Ear (n=12; 5,56%) | Accessory auricle  Pre-auricular tag  Pre-auricular sinus  Unilateral dysplastic ear  Malformation of the ear NOS | 3 (1,39)  3 (1,39)  2 (0,93)  1 (0,46)  3 (1,39) |
| Eye (n=2; 0,93%) | Aniridia  Corneal pigmentation | 1 (0,46)  1 (0,46) |
| Skin (n=3; 1,39%) | Sacral dermal cyst  Haemangioma chest  Haemangioma face | 1 (0,46)  1 (0,46)  1 (0,46) |
| Renal^‡^ (n=17; 7,87%) | Unilateral multi-cystic kidney disease  Renal dysplasia NOS  Accessory kidney  Duplex renal system  Duplicate ureter  Horseshoe kidney  Renal cyst  Single kidney | 7 (3,24)  4 (1,85)  1 (0,46)  1 (0,46)  1 (0,46)  1 (0,46)  1 (0,46)  1 (0,46) |

NOS – not otherwise specified

ⴕ includes uni- and bilateral, fingers and/or toes

‡ diagnosed on antenatal ultrasound

**Supplementary Table 4, Congenital Disorders visible on external examination at birth missed in the Pregnancy Exposure Registry**

|  | **Total** | | **Late diagnosis** | **FMU Astraia database** | |
| --- | --- | --- | --- | --- | --- |
| **Organ system (total)** | **Livebirth (NND)** | **Stillbirth** | **Livebirth (all)** | **Livebirth (NND)** | **Stillbirth** |
| Chromosomal (16)  Gastrointestinal (5)  Genitourinary (8) ^ⴕ^  Musculoskeletal (7)  Clefts (1)  Amniotic band (1)  Congenital syphilis (1)  Central Nervous System (6)  Cardiac^§^ (14)  Other (6)  Congenital diaphragmatic hernia  Capillary malformation | 13  4  8  4 (1)  1  1  0  5 (1)  14 (1)  1  1  4 | 3  1  0  3  0  0  1  1  0  0  0  0 | 13 (Down Syndrome)  3 (2 arm; 1 malrotation bowel)  8 (6 hypospadias; 2 dsd)  2 (1 congenital dislocation of the hip; 1 syndactyly)  2 (isolated cleft palate)^‡^  0  0  3 (1 cervical meningocoele; 1 craniosynestosis; 1 macrocephaly)  13 (1 coarctation aorta; 2 NOS; 2 complex cardiac; 1 TAPVD; 7 TOF)  1  1  4 | 0  1 (GIT NOS)  0  2(1) (Skeletal Defect)  1 (cleft lip and palate)  1  0  2 (1) ventriculomegaly  1(1) NOS  0  0  0 | 3 (1 Trisomy 18; 2 chromosomal abnormality NOS)  1 (omphalocoele)  0  3 (2 Skeletal Defect; 1 skeletal NOS)  0  1  1 ventriculomegaly  0  0  0  0 |
| Total (65) | 56 (3) | 9 | 48 | 8 (3) | 9 |

arm – anorectal malformation; dsd – disorders of sexual development; FMU – Fetal Medicine Unit; GIT – gastrointestinal tract; NND – neonatal death; NOS – not otherwise specified; TAPVD – total anomalous pulmonary venous drainage; TOF – Tetralogy of Fallot

ⴕ undescended testes excluded

‡ not included in totals as not visible in external examination at birth

§ cyanotic and critical cardiac defects

**Supplementary Table 5, Congenital Heart Disease**

| **Congenital Heart Disease visible on external neonatal examination at birth** | | | | | | | | |
| --- | --- | --- | --- | --- | --- | --- | --- | --- |
|  | **PER** | | | | **Inclusive** | | | |
|  | **Prevalence/1000 births** | **Total (NND)** | **Live birth (NND)** | **Still Birth** | **Prevalence/1000 births** | **Total (NND)** | **Live birth (NND)** | |
| Tetralogy of Fallot | 0,12/1000 | 4 (2) | 4 (2) | 0 | 0,34/1000 | 11 (2) | 11 (2) | |
| Total anomalous pulmonary venous drainage | 0,06/1000 | 2 | 2 | 0 | 0,09/1000 | 3 | 3 | |
| Complex cardiac | 0,09/1000 | 3 (3) | 3 (3) | 0 | 0,15/1000 | 5 (3) | 5 (3) | |
| Cardiac NOS | 0,03/1000 | 1 | 1 | 0 | 0, 21/1000 | 4 (1) | 4 (1) | |
| Hypoplastic left heart | 0,03/1000 | 1 | 1 | 0 | 0,03/1000 | 1 | 1 | |
| Coarctation of the aorta |  | 0 | 0 | 0 | 0,03/1000 | 1 | 1 | |
| Congenital heart block | 0,03/1000 | 1 | 1 | 0 | 0,03/1000 | 1 | 1 | |
| Total | 0,37/1000 | 12 (5) | 12 (5) | 0 | 0,80/1000 | 26 (6) | 26 | |
| **Acyanotic Congenital Heart Disease not visible on external neonatal examination at birth** | | | | | | | | |
|  | **PER** | | | | **Including late diagnosis** | | | |
|  | Prevalence/1000 births | Total (NND) | Live birth (NND) | Still Birth | Prevalence/1000 births | Total | Live birth (late) | Median age at dx (days) (IQR) |
| Dextrocardia | 0,03/1000 | 1 | 1 | 0 | 0,06/1000 | 2 | 2 | 14 |
| ASD | 0,06/1000 | 2 (1) | 2 (1) | 0 | 0,25/1000 | 8 (1) | 6 | 51 (12 – 80) |
| VSD | 0,15/1000 | 5 | 5 | 0 | 0,52/1000 | 17 | 12 | 60 (0 – 136) |
| AVSD | 0,03/1000 | 1 | 1 | 0 | 0,15/1000 | 5 | 4 | 90 (90 – 210) |
| anomalous left coronary artery from the pulmonary artery | 0,03/1000 | 1 | 1 | 0 | 0,03/1000 | 1 | 0 | 0 (at birth) |
| PS | 0,03/1000 | 1 | 1 | 0 | 0,22/1000 | 7 | 6 | 39,5 (6,5 – 69,5) |
| ASD + PS |  | 0 | 0 | 0 | 0,09/1000 | 3 | 3 | 150 (42 – 180) |
| PDA at term | 0,03/1000 | 1 | 1 | 0 | 0,03/1000 | 1 | 0 | 60 |
| ASD + PDA |  | 0 | 0 | 0 | 0,09/1000 | 3 | 3 | 41 (13 – 240) |
| Aortopulmonary window |  | 0 | 0 | 0 | 0,03/1000 | 1 | 1 | 49 |
| Congenitally Corrected Transposition of the Great Arteries |  | 0 | 0 | 0 | 0,03/1000 | 1 | 1 | 660 |
| Total | 0,37/1000 | 12 (1) | 12 (1) | 0 | 1,51/1000 | 49 (1) | 38 |  |
| Overall total | 0,74/1000 | 24 (6) | 24 (6) | 0 | 2,43/1000 | 75 | 64 |  |

ASD – atrial septal defect; AVSD – atrio-ventricular septal defect; PDA – patent ductus arteriosus; PS – pulmonary stenosis; VSD – ventricular septal defect
