# Supplementary material for: Population-based prevalence of congenital defects in a routine sentinel-site based surveillance system in the Western Cape, South Africa
Source: Birth Defects Res. Author manuscript; Available in PMC 2025 Jan 15. (PMC11733423; doi:10.1002/bdr2.2388)
Supplement: 2 [file NIHMS2043832-supplement-2.pdf]

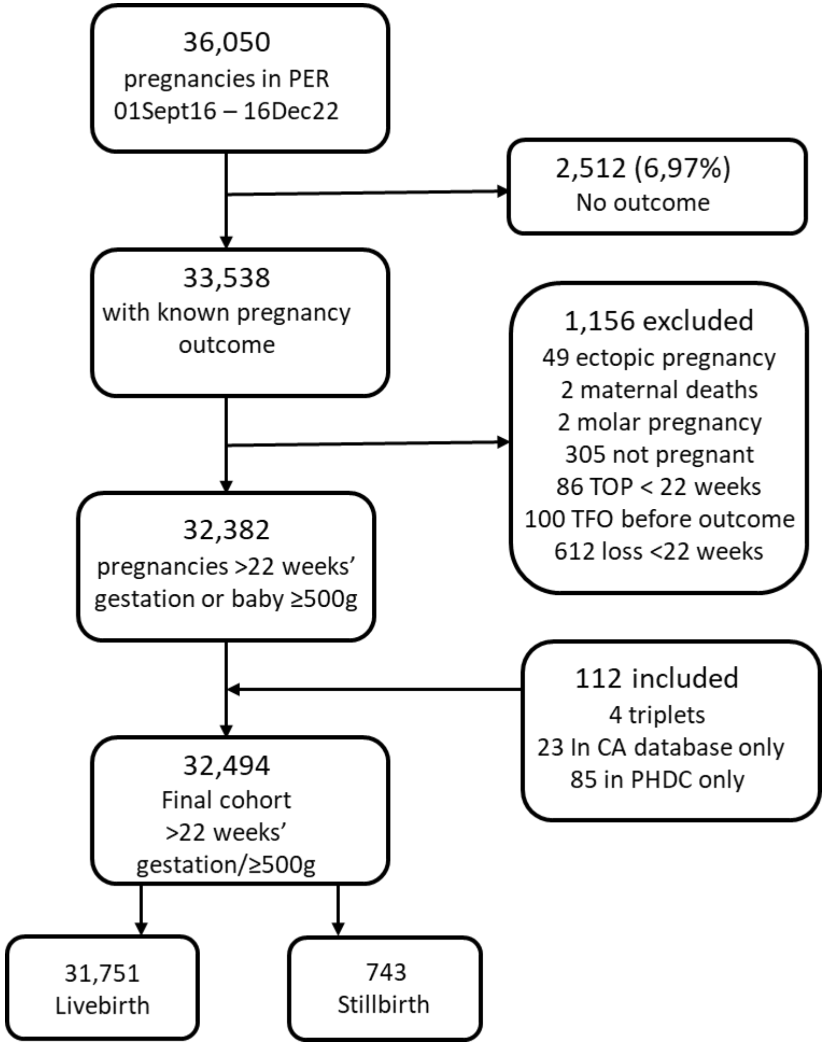

Supplementary Figure 1: Cohort Definition

CD – congenital disorder; PER – Pregnancy Exposure Registry; PHDC – Provincial Health Data Centre; TFO – transferred out of region; TOP – termination of pregnancy
